# Bedside Calculation of Energy Expenditure Does Not Guarantee Adequate Caloric Prescription in Long-Term Mechanically Ventilated Critically Ill Patients: A Quality Control Study

**DOI:** 10.1100/2012/909564

**Published:** 2012-05-15

**Authors:** Elisabeth De Waele, Herbert Spapen, P. M. Honoré, Sabrina Mattens, Thomas Rose, Luc Huyghens

**Affiliations:** ^1^Department of Intensive Care Medicine, University Hospital, Vrije Universiteit Brussel, Laarbeeklaan 101, 1090 Brussels, Belgium; ^2^Department of Dietetics, University Hospital, Vrije Universiteit Brussel, 1090 Brussels, Belgium

## Abstract

Nutrition is essential in critically ill patients, but translating caloric prescriptions into adequate caloric intake remains challenging. Caloric prescriptions (P), effective intake (I), and caloric needs (N), calculated with modified Harris-Benedict formulas, were recorded during seven consecutive days in ventilated patients. Adequacy of prescription was estimated by P/N ratio. I/P ratio assessed accuracy of translating a prescription into administered feeding. I/N ratio compared delivered calories with theoretical caloric needs. Fifty patients were prospectively studied in a mixed medicosurgical ICU in a teaching hospital. Basal and total energy expenditure were, respectively, 1361 ± 171 kcal/d and 1649 ± 233 kcal/d. P and I attained 1536 ± 602 kcal/d and 1424 ± 572 kcal/d, respectively. 24.6% prescriptions were accurate, and 24.3% calories were correctly administered. Excessive calories were prescribed in 35.4% of patients, 27.4% being overfed. Caloric needs were underestimated in 40% prescriptions, with 48.3% patients underfed. Calculating caloric requirements by a modified standard formula covered energy needs in only 25% of long-term mechanically ventilated patients, leaving many over- or underfed. Nutritional imbalance mainly resulted from incorrect prescription. Failure of “simple” calculations to direct caloric prescription in these patients suggests systematic use of more reliable methods, for example, indirect calorimetry.

## 1. Background

Delivering a correct amount of calories to critically ill patients is considered to be of cardinal importance [1, 2]. Indeed, inadequate nutrition (i.e., under- or overfeeding) in this population has distinct effects on immuno-inflammatory pathways, is associated with increased morbidity, and may impair survival [3, 4]. Underfeeding disturbs the regeneration of respiratory epithelium and causes respiratory muscle dysfunction [5] which may prolong ventilator dependence [6]. Even when present subclinically, it is responsible for reduced superficial and deep wound healing [7]. Also, failure to provide more than 25% of recommended calories significantly increases the risk of bloodstream infection [8]. In contrast, overfeeding is more likely to cause metabolic disturbances (hypertriglyceridemia, hyperglycemia, and azotemia) but may also be at the origin of organ (hepatic, respiratory) dysfunction [9, 10].

Still, discrepancies between theoretical energy requirements and actual delivery of nutrition in intensive care unit (ICU) patients are more rule than exception [10, 11]. Moreover, studies evaluating whether quality of nutrition matches current ICU feeding guidelines remain scarce, particularly in specific patients such as those receiving prolonged mechanical ventilation [12].

We therefore prospectively studied whether feeding prescriptions were translated into adequate caloric intake within the scope of a “real-life,” guideline-oriented nutritional approach in a population of mechanically ventilated critically ill patients. 

## 2. Methods

The study was approved by the hospital's Ethical Committee. Due to its observational nature, the need for informed consent was waived.

During a 4-month period, we included all patients older than 18 years admitted to our medicosurgical ICU when intubated and expected to receive mechanical ventilation for at least seven days. Patients were ventilated in pressure- or volume-controlled modes under continuous analgesic sedation with remifentanil and midazolam. Whenever possible, intravenous dextrose infusions were avoided. Insulin was infused to maintain a target blood glucose level of 80–110 mg/dL. All subjects received enteral and/or parenteral feeding, as part of their standard treatment. Feeding was provided according to a dedicated nutritional care plan. The used protocol closely reflected current evidence-based, easy-to-use feeding algorithms indicating amount, composition and route of delivery.

Gender, age, weight, height, and type of pathology were recorded at study entry. For obese patients, optimal caloric intake was calculated for a theoretical weight corresponding to a body mass index of 30 kg/m² [13]. Total caloric need (N, kcal/d) was assessed by multiplying basal energy expenditure, calculated with a modified Harris-Benedict equation as follows: male: resting energy expenditure (REE) (kcal/d) = 66.47 + 13.75 (wt) + 5.003 (ht) − 6.755 (a) (years); female: REE (kcal/d) = 655.1 + 9.563 (wt) + 1.850 (ht) − 4.676 (a) (years), where wt denotes weight, ht represents height and a refers to age [14], which was, daily adjusted for weight and stress. For uncomplicated and complicated surgery, a stress factor of, respectively, 1.1 and 1.3 was used. Fractures and polytrauma were given, respectively, 1.1 and 1.3. Patients with an uncomplicated infection received a correction factor of 1.1, but sepsis was attributed 1.3.

Attending ICU physicians, unaware of the study, based their daily caloric prescriptions on the expert-recommended 25 kcal/kg/d regimen [15]. For seven consecutive days, prescriptions (P, kcal/d) and effective intake (I, kcal/d) were recorded. A dedicated nutrition team measured caloric requirements with the "stress-adapted" Harris-Benedict formulas and estimated correctness of the prescription by calculating the P/N ratio. The accuracy of translating a prescription into really administered feeding was assessed by the I/P ratio. Finally, the I/N ratio was calculated to compare the amount of delivered calories with the theoretical caloric need. The latter was set at 100%, and both prescriptions and actual feeding were expressed as proportional to this percentage. A prescription was considered to be adequate when it covered 90 to 110% of total caloric need. Prescriptions not attaining 90% were considered to be “underestimated” whilst those exceeding 110% were said to be “overestimated.”

Statistical analysis used SPSS 12.0 for Windows (Chicago, IL, USA). Results were expressed as means ± standard deviation and medians (range). Means between groups were compared with the Student's *t*-test. Statistical significance was accepted at a *P* value < 0.05.

## 3. Results

579 patients were admitted to the ICU during the study period. 231 subjects were mechanically ventilated. Of the 81 patients meeting enrolment criteria, thirty-one were excluded from analysis. Reasons for exclusion were extubation before day 7 (*n* = 14), tracheostomy (*n* = 6), do-not-resuscitate order given (*n* = 3), and death (*n* = 8). Finally, fifty patients, 28 males and 22 females, representing a total of 350 nutrition days, were studied. Mean age was 65 (range 34–84) years. Mean body weight and length were, respectively, 76.9 ± 18.3 kg and 169.6 ± 9.7 cm. Twelve patients were obese. Mean APACHE II score was  28 ± 12. Mean resting energy expenditure for all studied patients was 1361 ± 171 kcal/d. Thirteen patients were allocated a stress factor 1.1, 18 patients received 1.2, and 19 subjects were assigned a factor 1.3. This resulted in a mean total energy expenditure of 1649 ± 233 kcal/d.

**Figure 1:**
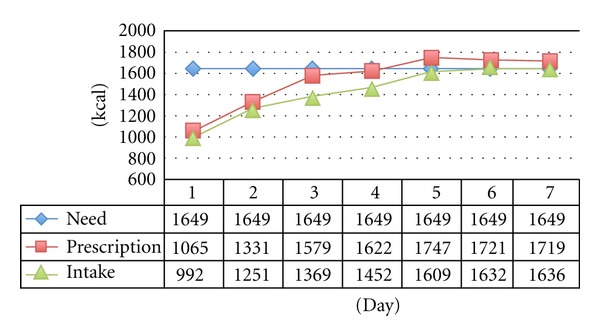
Evolution in time of caloric need, prescription and intake over the study period.

The mean daily amount of calories prescribed during the 350 study days reached 1536 ± 602 kcal. In average, 1424 ± 572 kcal/d were actually delivered to the patient. P and I varied with time (Figure 1). During the study period, median P/N ratio was 0.97 (range 0.0–1.80), mean I/N ratio 0.91 (range 0.0–1.84), and mean I/P ratio, or delivery rate, 0.95 (range 0.08–7.20).

24.6% of the 350 nutritional prescriptions correctly estimated the need. In 40.0% of cases, nutritional needs were insufficiently covered. Overestimation occurred in the remaining 35.4%.

**Figure 2:**
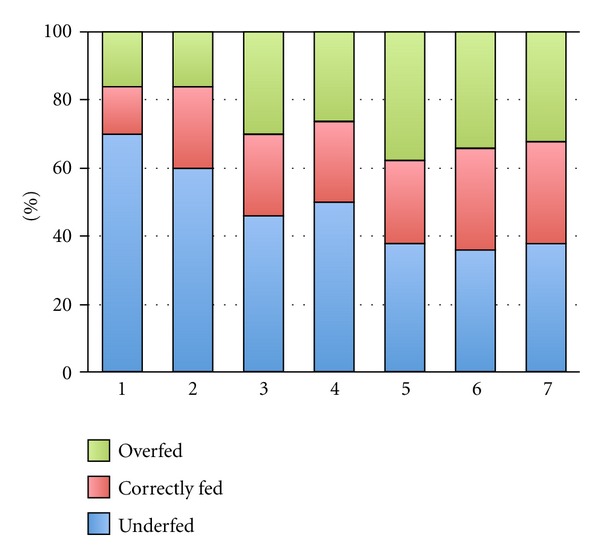
Feeding status per day.

Using similar cutoff percentages to evaluate effective feeding, patients were, respectively, correctly, over-, or underfed in 24.3%, 27.4%, and 48.3% of the nutrition days. Underfeeding was more frequent on the first as compared to the next 6 ventilation days (Figure 2). In fact, either no nutritional prescription was found in the medical records or feeding was started without a formal prescription.

The amount of effectively administered calories varied with time. Caloric prescription resulted in accurate delivery in 56.0% of cases. However, effective feeding was not met in 32.6% of prescriptions, and in 9.14% actual feeding surpassed the prescribed amount by more than 10%.

**Figure 3:**
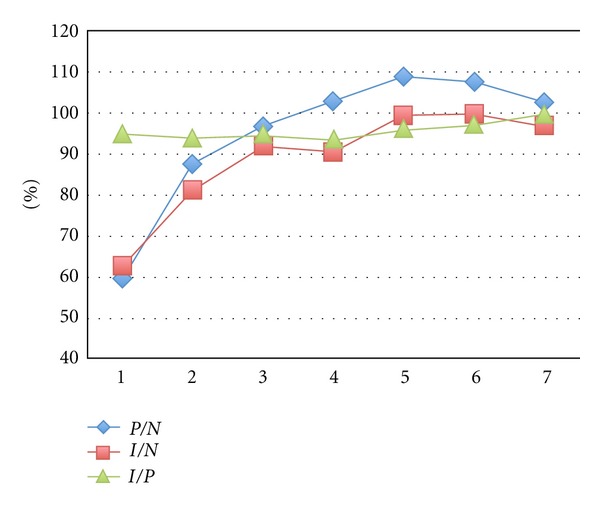
Evolution in time of median P/N, I/N, and I/P ratio.

Mean P/N ratio rose from 59.3 on day 1 to 102.7 on day 7, whilst the median I/N ratio increased from 62.6 to 97 during the same period. Thus, both estimation of needs and actually administered feeding improved with time. The delivery rate did not vary significantly during the observation period (Figure 3).

## 4. Discussion

Nutrition is an indispensable part of overall treatment in critically ill patients. Fundamental goals of nutritional support in the ICU are to meet energy requirements of (hyper)metabolic processes, to prevent nutrient deficiencies and to minimize protein catabolism. Whilst inadequate nutrition, in general, is known to significantly compromise outcome in the critically ill, its unwarranted effects may be even more pronounced in mechanically ventilated patients. Indeed, animals submitted to fasting or receiving long-term hypocaloric feeding in both aerobe and anaerobe conditions experienced muscle decay and dysfunction which incompletely recovered after realimentation [16, 17]. It is conceivable that these findings may translate into enhanced and/or prolonged respiratory failure and thus longer ventilator-dependency in humans.

Throughout the literature, most discussion regarding nutrition in mechanically ventilated patients is focused on type, composition, and caloric/nitrogen content of available feeding liquids. However, evaluating adequate, that is, correct and effective, feeding in this population remains challenging. The present study confirms that energetic requirements in critically ill, mechanically ventilated patients differ considerably in accordance to the severity of the underlying pathology. In general, energetic needs were well anticipated by the attending physicians, yet variations were large. 25% of the caloric prescriptions were correct, but a stunning 75% resulted in under- or overfeeding. Effective administration of calories followed the same trend as the prescription. However, energetic requirements were met in only 24% of the feeding days. The discrepancy between caloric prescription and intake caused underfeeding in nearly half and overfeeding in 27% of the study days. Our findings also highlighted that nutritional prescription was fairly well translated into effective feeding in the majority of patients but that extreme variations in intake/prescription ratio (up to 720% !) could occur. A possible explanation is that oral nutrition orders were executed without being recorded in the patient's files. Our results, demonstrating (a) > 90% I/P and P/N ratio after 72 hours, are in agreement and even better than those reported recently by Quenot et al. [18]. However, these authors only studied enteral nutrition aiming at a minimal caloric supply of 25 kcal/kg/day and did not calculate stress-adjusted energy requirements. Interestingly, they found that the I/P ratio was significantly influenced by gastric residual volume measurement [18].

Thirty years ago, Driver and LeBrun described iatrogenic malnutrition in more than 80% of mechanically ventilated patients [19]. Although nutrition policy in the ICU has considerably improved since, de Jonghe et al. recently reported that energetic needs still remained inadequately covered in more than 20% of ICU patients [11]. McClave et al. reported correct estimation of energetic needs in 29%, overestimation in 58%, and underestimation in 12% of cases. Fifty-eight percent of the patients were overfed, and 39% received too much calories. Correct feeding was provided in 25% of nutrition days which corresponds very well with the 24% incidence observed in our study [10]. Kan et al. reported adequate feeding in 37% and overfeeding in 35% critically ill ventilated patients [6], which also matches our results. Interestingly, physicians in our ICU tended to prescribe fewer calories than needed whereas in the above-mentioned studies caloric needs were generally overestimated, resulting in overfeeding. Traditionally, it is accepted that overfeeding produces higher CO_2_ blood levels resulting in increased minute ventilation [20]. Nonetheless, McClave et al. found an inverse relationship between the amount of nutrition and minute ventilation in mechanically ventilated subjects. However, this study is different from ours since patients were given only enteral feeding [10].

Nutritional care of critically ill patients is complicated. Patients form heterogeneous groups that are prone to significant and continuous metabolic fluctuations induced by type, severity, and evolution of the disease process. In addition, confounding variables such as over- or underweight, resuscitation edema, and concomitant medication (e.g., sedation) may all hamper correct estimation of metabolic demands [21, 22]. Indirect calorimetry definitely is the gold standard for determination of resting energy expenditure, but, when not available, specific prediction equations have up to now been widely accepted as an alternative [15]. To account for levels of disease or injury severity and complications, the so-called stress factors have been introduced. These corrective factors were obtained by comparing direct calorimetry measurements between hospitalized patients and healthy volunteers and are, by definition, arbitrary in the critically ill. Of note is that specific correction factors for ventilated patients are scarce. Casati et al. multiplied basal energy expenditure by 1.20, 1.28, or 1.50 in, respectively, nonsurgical/nonseptic conditions, complicated surgery, and severe infection/multiple trauma [23]. Cheng et al. applied a stress factor of 1.25 in all mechanically ventilated patients [14]. Kan et al. reported that at least 120% of resting energy requirement had to be administered to meet caloric needs in ventilated patients [6]. We used a modified Harris-Benedict equation attempting to anticipate on daily stress and injury events in a particular patient. Though considered to be an unreliable predictor of caloric needs in critically ill patients [24, 25], the Harris-Benedict formula proved to be relatively accurate in this population when a factor of 1.1 was multiplied to the equation [26]. Moreover, in a cohort of mechanically ventilated ICU patients, a modified Harris-Benedict equation (i.e., multiplied by a factor 1.2 and incorporating actual body weight) was found to be within 15% of measured energy expenditure determined by indirect calorimetry [27].

Our study has several shortcomings. First, calculations of caloric intake did not account for caloric content of eventually administered dextrose-containing infusions. Second, caloric requirements ideally should be measured by indirect calorimetry. Feeding near-target energy requirements based on repeated calorimetric measurements was associated with lower hospital mortality [28]. However, indirect calorimetry is not widely available or affordable. It is time consuming, requires dedicated equipment and staff, and only reflects the short-time window during which the patient is studied. Some guidelines for nutrition support in mechanically ventilated, critically ill adult patients do not even recommend its use [29]. Our approach using “corrected” Harris-Benedict equations to calculate energy requirements was motivated by the wide acceptance of this easy method for bedside assessment of nutritional therapy in ICU patients and is supported by the ESPEN guidelines on parenteral nutrition in an ICU setting [30]. Third, we did not assess outcome in our patients. Major reasons are the small study sample and the lack of correction for baseline differences in severity of disease. Moreover, the feeding protocol used in this study does not allow coping with the recently roused controversy regarding parenteral nutrition and outcome [31, 32]. Finally, the observational nature of our study obviously has inherent limitations though not altering the key message that basic directives obtained in well-controlled research settings do not easily translate in providing “quality" nutrition in daily ICU practice. 

## 5. Conclusion

This prospective quality control study demonstrated an important dissimilarity between the amount of calories prescribed according to current nutritional guidelines and the caloric need calculated by a stress-corrected Harris-Benedict equation in critically ill mechanically ventilated patients. This was due to inadequate prescription and, to a lesser degree, to inappropriate conversion of correct prescriptions into “true” feeding. Repeated evaluation of caloric needs and administration using best evidence measurement tools and continuous feedback to all involved health care workers are critical issues for providing optimal nutritional care in these patients. In this context, a dedicated nutrition support team may play an important role [33]. Our observations add support to a more systematic use of indirect calorimetry in long-term mechanically ventilated patients.

## Abbreviations

P: Prescriptions
I: Intake (effective)
N: Needs (caloric)
ICU: Intensive care unit.

## References

[1] Jolliet P, Pichard C, Biolo G (1998). Enteral nutrition in intensive care patients: a practical approach. *Intensive Care Medicine*.

[2] American Society for Parenteral and Enteral Nutrition (1993). Guidelines for the use of parenteral and enteral nutrition in adults and pediatric patients. *Journal of Parenteral and Enteral Nutrition*.

[3] Harbige LS (1996). Nutrition and immunity with emphasis on infection and autoimmune disease. *Nutrition and Health*.

[4] Strack van Schijndel RJM, Weijs PJM, Koopmans RH, Sauerwein HP, Beishuizen A, Girbes ARJ (2009). Optimal nutrition during the period of mechanical ventilation decreases mortality in critically ill, long-term acute female patients: a prospective observational cohort study. *Critical Care*.

[5] Askanazi J, Weissman C, Rosenbaum SH, Hyman AI, Milic-Emili J, Kinney JM (1982). Nutrition and the respiratory system. *Critical Care Medicine*.

[6] Kan MN, Chang HH, Sheu WF, Cheng CH, Lee BJ, Huang YC (2003). Estimation of energy requirements for mechanically ventilated, critically ill patients using nutritional status. *Critical Care*.

[7] Silberman H (1989). *Parenteral and Enteral Nutrition*.

[8] Rubinson L, Diette GB, Song X, Brower RG, Krishnan JA (2004). Low caloric intake is associated with nosocomial bloodstream infections in patients in the medical intensive care unit. *Critical Care Medicine*.

[9] Klein CJ, Stanek GS, Wiles CE (1998). Overfeeding macronutrients to critically ill adults: metabolic complications. *Journal of the American Dietetic Association*.

[10] McClave SA, Lowen CC, Kleber MJ (1998). Are patients fed appropriately according to their caloric requirements?. *Journal of Parenteral and Enteral Nutrition*.

[11] de Jonghe B, Appere-De-Vechi C, Fournier M (2001). A prospective survey of nutritional support practices in intensive care unit patients: what is prescribed? What is delivered?. *Critical Care Medicine*.

[12] Frutiger A, Moreno R, Thijs L, Carlet J (1998). A clinician's guide to the use of quality terminology. *Intensive Care Medicine*.

[13] Barak N, Wall-Alonso E, Sitrin MD (2002). Evaluation of stress factors and body weight adjustments currently used to estimate energy expenditure in hospitalized patients. *Journal of Parenteral and Enteral Nutrition*.

[14] Cheng CH, Chen CH, Wong Y, Lee BJ, Kan MN, Huang YC (2002). Measured versus estimated energy expenditure in mechanically ventilated critically ill patients. *Clinical Nutrition*.

[15] Kreymann KG, Berger MM, Deutz NEP (2006). ESPEN guidelines on enteral nutrition: intensive care. *Clinical Nutrition*.

[16] Nishio ML, Jeejeebhoy KN (1992). Effect of malnutrition on aerobic and anaerobic performance of fast- and slow-twitch muscles of rats. *Journal of Parenteral and Enteral Nutrition*.

[17] de la Torre AM, Madapallimattam A, Cross A, Armstrong RL, Jeejeebhoy KN (1993). Effect of fasting, hypocaloric feeding, and refeeding on the energetics of stimulated rat muscle as assessed by nuclear magnetic resonance spectroscopy. *Journal of Clinical Investigation*.

[18] Quenot JP, Plantefeve G, Baudel JL (2010). Bedside adherence to clinical practice guidelines for enteral nutrition in critically ill patients receiving mechanical ventilation: a prospective, multi-centre, observational study. *Critical Care*.

[19] Driver AG, LeBrun M (1980). Iatrogenic malnutrition in patients receiving ventilatory support. *Journal of the American Medical Association*.

[20] Amene PC, Sladen RN, Feeley TW, Fisher R (1987). Hypercapnia during total parenteral nutrition with hypertonic dextrose. *Critical Care Medicine*.

[21] Weissman C, Kemper M, Askanazi J (1986). Resting metabolic rate of the critically ill patient: measured versus predicted. *Anesthesiology*.

[22] Weissman C, Kemper M, Elwyn DH (1986). The energy expenditure of the mechanically ventilated critically ill patient: an analysis. *Chest*.

[23] Casati A, Colombo S, Leggieri C, Muttini S, Capocasa T, Gallioli G (1996). Measured versus calculated energy expenditure in pressure support ventilated ICU patients. *Minerva Anestesiologica*.

[24] Walker RN, Heuberger RA (2009). Predictive equations for energy needs for the critically ill. *Respiratory Care*.

[25] Frankenfield D, Hise M, Malone A, Russell M, Gradwell E, Compher C (2007). Prediction of resting metabolic rate in critically Ill adult patients: results of a systematic review of the evidence. *Journal of the American Dietetic Association*.

[26] Boullata J, Williams J, Cottrell F, Hudson L, Compher C (2007). Accurate determination of energy needs in hospitalized patients. *Journal of the American Dietetic Association*.

[27] Alexander E, Susla GM, Burstein AH, Brown DT, Ognibene FP (2004). Retrospective evaluation of commonly used equations to predict energy expenditure in mechanically ventilated, critically ill patients. *Pharmacotherapy*.

[28] Singer P, Anbar R, Cohen J (2011). The tight calorie control study (TICACOS): a prospective, randomized, controlled pilot study of nutritional support in critically ill patients. *Intensive Care Medicine*.

[29] Heyland DK, Dhaliwal R, Drover JW, Gramlich L, Dodek P (2003). Canadian clinical practice guidelines for nutrition support in mechanically ventilated, critically ill adult patients. *Journal of Parenteral and Enteral Nutrition*.

[30] Singer P, Berger MM, van den Berghe G (2009). ESPEN guidelines on parenteral nutrition: intensive care. *Clinical Nutrition*.

[31] Kutsogiannis J, Alberda C, Gramlich L (2011). Early use of supplemental parenteral nutrition in critically ill patients: results of an international multicenter observational study. *Critical Care Medicine*.

[32] Casaer MP, Mesotten D, Hermans G (2011). Early versus late parenteral nutrition in critically ill adults. *The New England Journal of Medicine*.

[33] Frost P, Bihari D (1997). The route of nutritional support in the critically Ill: physiological and economical considerations. *Nutrition*.

